# Bioluminescence-Driven Optogenetics

**DOI:** 10.3390/life10120318

**Published:** 2020-11-28

**Authors:** Macià Sureda-Vives, Karen S. Sarkisyan

**Affiliations:** 1 Synthetic Biology Group, MRC London Institute of Medical Sciences, London W12 0NN, UK; m.sureda-vives17@imperial.ac.uk; 2 Institute of Clinical Sciences, Faculty of Medicine, Imperial College London, London W12 0NN, UK; 3 Shemyakin-Ovchinnikov Institute of Bioorganic Chemistry, Russian Academy of Sciences, Moscow 117997, Russia

**Keywords:** bioluminescence, optogenetics, BRET, synthetic biology, luciferase, luciferin, luminopsin, photosensitiser, light-based communication

## Abstract

Bioluminescence-based technologies are among the most commonly used methods to quantify and visualise physiology at the cellular and organismal levels. However, the potential of bioluminescence beyond reporter technologies remains largely unexplored. Here, we provide an overview of the emerging approaches employing bioluminescence as a biological light source that triggers physiological events and controls cell behaviour and discuss its possible future application in synthetic biology.

## 1. Introduction

Bioluminescent organisms derive energy from enzymatic reactions to generate photons. The process relies on small molecules called luciferins that can reach light-emitting electronic states when catalytically oxidised. Across the tree of life, numerous enzymes, known as luciferases, have evolved to control the oxidation of individual luciferins. As a result, nature holds a diverse pool of light-emitting reactions with distinct wavelengths, cofactor dependencies, and other properties [1,2]. For most bioluminescent systems, the enzymes catalysing luciferin biosynthesis are still unknown, with the exception of the pathways of bacteria and fungi [3]. These two fully-encodable systems can be transferred to prokaryotic and eukaryotic hosts to generate organisms with self-sustained light emission (Table 1) [4,5,6,7,8]. Other known bioluminescence systems require exogenous addition of synthetic luciferin when heterologously expressed.

The utility of genetic elements with the intrinsic ability to produce light, an easily measurable signal, is conspicuous. Resolving the structures of the first luciferase-luciferin pairs was enough for bioluminescence to become a valuable reporter technology. It is now routinely used in gene expression assays, cell physiology, immunoassays, food analysis, drug screenings, and environmental monitoring [9,10]. More recently, the development of sensitive digital cameras and bioluminescent tools that can be detected in tissues of intact animals turned bioluminescence into a powerful *in vivo* imaging technology [11,12]. 

**Figure 1:**
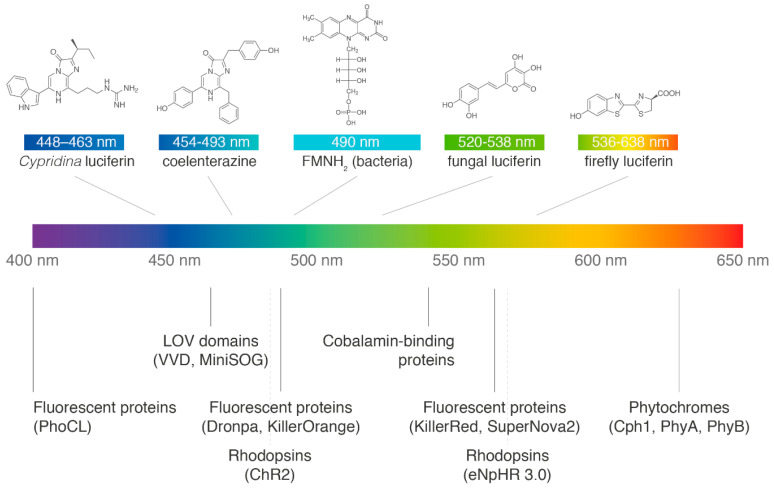
Overview of bioluminescence systems where the structure of the luciferin and at least one luciferase are known and available (upper part), and the main available groups of optogenetic tools (lower part). The approximate location of the described systems is shown on the visible spectrum, based on light emission spectra of the native luciferins, and excitation spectra of the optogenetic tools. PhoCL, Photocleavable protein; VVD, Vivid; miniSOG, mini Singlet Oxygen Generator; ChR2, Channelrhodopsin-2; eNpHR 3.0, enhanced *Natronomonas* halorhodopsin; Cph1, Cyanobacterial phytochrome-1; PhyA/B, Phytochrome A/B.

Traditionally, luciferases have not been considered beyond their role as reporter proteins, yet light offers certain advantages over chemical modulators for controlling biological systems. Light signals are inert, can be delivered with high spatiotemporal resolution, and allow fast-reverting kinetics [13]. In the field of optogenetics, numerous light-sensing proteins from nature have been repurposed to interrogate and engineer cellular processes with external light illumination [14,15]. However, coupling bioluminescent proteins to these light-inducible systems has not been considered until very recently, despite the fact that the existing array of optogenetic and bioluminescent tools has the potential for engineering biological circuits that emit, sense, and respond to light in most of the visible and near-infrared parts of the spectrum (Figure 1). This review aims to provide a summary of the initial achievements in deploying bioluminescence to control cellular processes.

## 2. Energy Transfer to Fluorescent Proteins 

The first examples of artificial coupling of bioluminescent proteins to light-absorbing ones have been inspired by a naturally occurring mechanism for modulation of the colour of bioluminescence. Some species of cnidarians, such as *Aequorea victoria*, and bacteria, such as *Vibrio fischeri*, evolved non-homologous fluorescent proteins to interact with their bioluminescence systems, shifting the colour of the emitted light from blue to green or yellow [19]. This phenomenon is based on a non-radiative mechanism called bioluminescence resonance energy transfer (BRET). In this process, the energy stored in the excited state of the oxyluciferin is transferred to the chromophore of an interacting fluorescent protein which then emits a photon, typically of a longer wavelength [20]. Following this principle, several bioluminescent constructs based on a single luciferase have been engineered to re-emit bioluminescent light across the visible spectrum, from cyan to red [21,22,23,24,25]. The ability to alter the spectrum of a bioluminescent protein in such a controlled and modular manner makes deploying bioluminescent tools to control biological processes highly accessible.

## 3. Delivering Light to Optogenetic Tools

**Luminopsins.** Since the development of the first optogenetic tools, it has been a challenge to deliver light to proteins expressed in deep animal tissues, or pigmented plant tissues, without physically damaging the organism and inducing physiological responses or phototoxicity due to strong light illumination [26,27].

In neuroscience, photoactivatable ion channels, such as bacterial opsins, have conventionally been expressed in specific neuronal lineages to functionally interrogate brain circuitry using light. Typically, optical fibres are surgically implanted in the skull of animals, such as mice, to deliver light signals and control neuronal activity [28]. Despite the great success of these strategies, working with implanted optical fibres poses some technical impediments: for instance, the amount of neural structures that can be controlled simultaneously and their localization are limited by the number of fibres that can be introduced and the regions that can be accessed without damaging the brain [29]. Furthermore, light delivery is constrained by the absorption, scattering, and thermal sensitivity of biological tissues. The functional penetration of photons is restricted to 1 mm from the tip of the fibre [30], and the spectral window available for stimulation is reduced to red and near infrared light (600–1200 nm) [31,32]. Multiple approaches are being developed to non-invasively regulate neuronal activity using infra-red light, ultrasound, or small molecule administration [33,34,35].

**Figure 2:**
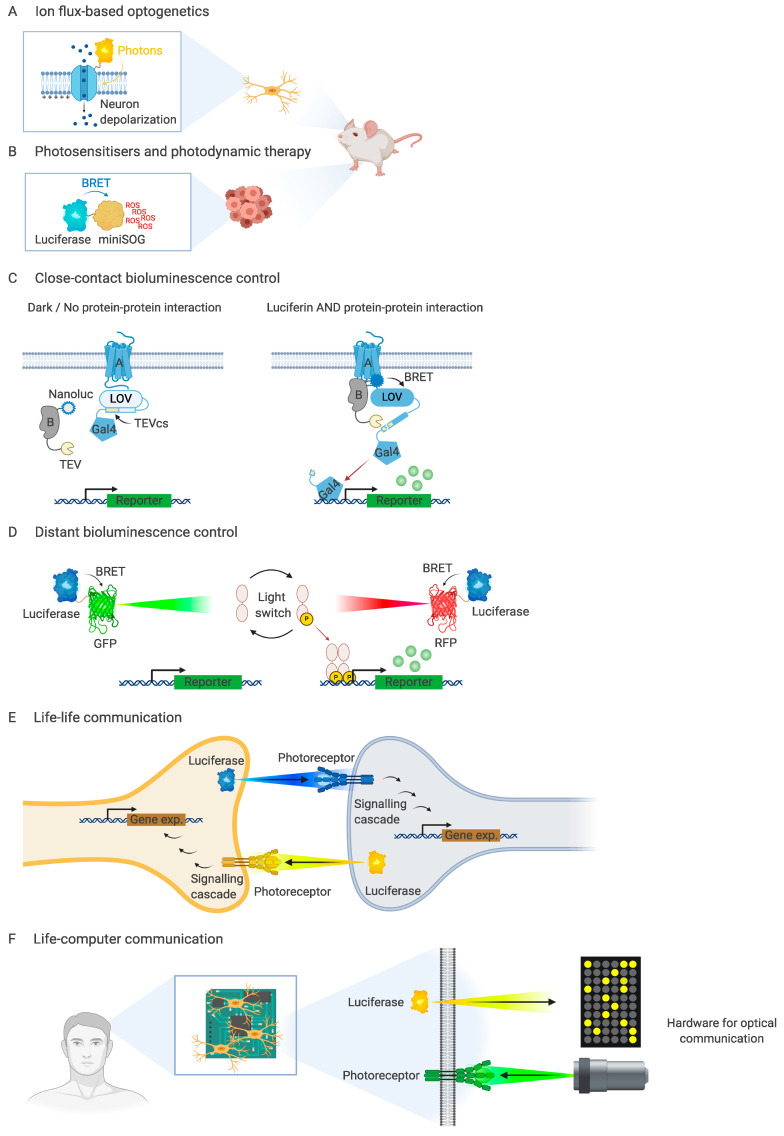
Summary of main bioluminescence applications in controlling cell processes and transferring information between molecules, genetic circuits, and systems (biological and digital). Self-illuminating optogenetic probes allow light-based regulation in vivo irrespective of their localization (**A**,**B**). The information transfer between light-emitting and light-sensing proteins can be programmed to operate either at short or long distances inside the cell (**C**,**D**), and thus, potentially, light could be harnessed to engineer life–life and life–computer communication (**E**,**F**).

These limitations have also been circumvented by physically coupling a luciferase and an opsin of compatible spectra via a peptide linker [36,37,38,39]. In such hybrid constructs, termed luminopsins, the light generated by the luciferase activates the proton channel as an external light source would do, depolarising the cell membrane (Figure 2A). This enables the dual control of neuronal activity with a single construct, either systemically upon luciferin administration, which can be simultaneously detected with bioluminescence imaging, or locally with high spatiotemporal precision using optic fibres [40].

In the last few years, the luminopsins toolbox expanded to include inhibitory and step-function luminopsins. Inhibitory luminopsins are composed of a luciferase and a light-sensitive ion pump that causes hyperpolarization, silencing neural spiking instead of triggering it [37,38,41]; step-function luminopsins include mutations in the luminal side of the opsin channels that result in slower-reverting kinetics and increased photosensitivity [39]. Moreover, they have been further optimised by including a neural membrane trafficking peptide to reduce their aggregation and increase their membrane expression [42].

All the reported constructs contain luciferase variants, whose substrate, coelenterazine, can cross the blood-brain barrier [37]. Neural firing was shown to be proportional to bioluminescence over a certain range of coelenterazine concentrations, and the potential off-target effects of bioluminescence, coelenterazine, coelenterazine oxidation products, and vehicle solutions, on neural activation have also been evaluated [43]. It has been demonstrated that only certain high doses of coelenterazine showed non-specific alteration of neural activity [44,45]. It is also noteworthy that the luciferase and the opsin do not need to be physically linked to each other for this activation to be possible and may be simply co-expressed [41,46].

Bioluminescent-OptoGenetics (BL-OG) is the term coined by the authors to refer to this neural control paradigm, and its contribution to neuroscience is already a tangible one [47]. It has been applied to identify the implication of motoneurons in mediating the therapeutic effects of moderate daily exercise after peripheral nerve injury [48] and to achieve the simultaneous inhibition of multiple structures in the hippocampus of epileptic rats to examine and block the neural networks involved in the disease [49]. Its combination with stem cell therapy has proven to be considerably successful, as neural precursors expressing luminopsins can be chronically stimulated after transplantation irrespective of their localization within the nervous system, which may change in time. As a result, it was possible to improve the motor functions in a Parkinson’s disease mice model [50]; to induce neural network repair in ischemic mice brains [51]; and to rehabilitate mice after severe spinal cord injury [52]. Importantly, these BL-OG based therapies can be prospectively evaluated for future clinical studies due to the apparent safety of coelenterazine.

**Photosensitisers.** The technical constraints of delivering light in vivo are also faced by other research areas, expanding the usefulness of luciferases as biological light sources. Genetically encodable photosensitisers, proteins that produce reactive oxygen species when illuminated with light, have been developed to controllably ablate specific cell populations or proteins [53,54]. They have been used in developmental biology and neuroscience [55,56,57,58,59], as well as in model systems of tumour treatment [60,61,62,63]. However, the requirement for light delivery constrains application of this technology to deep tissues or spatially distributed cell populations.

To allow for light delivery without optical fibres, a fusion of NanoLuc luciferase and the phototoxic flavoprotein miniSOG was designed, resulting in the first genetically encodable, self-illuminating photosensitiser [64] (Figure 2B). A single dose of luciferin was shown to kill 48% of human breast cancer cells stably expressing the construct. Remarkably, BRET-induced cytotoxic effects from NanoLuc-miniSOG construct in vitro were comparable to those observed using external illumination. Furthermore, it was demonstrated that targeting this construct to mitochondria with a subcellular localization tag increased its cytotoxicity up to 65% and specifically induced cell death via apoptosis as opposed to when targeted to the plasma membrane that caused necrosis [65,66].

The same approach is applicable to other photosensitisers, allowing us to select the level and mechanism of phototoxicity suitable for the intended application. For example, a fusion of the firefly luciferase to the photosensitiser KillerRed was shown to produce low levels of reactive oxygen species and to trigger a change in actin organisation when targeted to F-actin [67]. The available palette of genetically encodable photosensitisers and spectrally compatible but chemically orthogonal bioluminescence systems make it potentially possible to combine multiple self-illuminating photosensitizers in the same system.

## 4. Towards Complexity: Programming Intracellular and Cell-Cell Interactions with Light

In the applications above, luciferases were used as biological light sources simply to reach places in vivo that optical hardware could not access. Yet, the utility of bioluminescence in other than in vivo scenarios might be underestimated due to the tunability and efficiency of available electronic systems for in vitro optogenetics [68]. As sophistication of engineering of intracellular processes increases, more complex electronic systems are required, or they become powerless, such as when controlling spatially restricted intracellular events or genetically programmed molecular interactions.

For certain applications, proteins need to be modulated over specific periods of time in defined subcellular compartments or microdomains [69]. Their activity might have distinct downstream effects depending on the spatiotemporal activation within the cell [70]. This is particularly important in mechanistic studies of signalling pathways. For instance, eukaryotic cells use cAMP as a general secondary messenger molecule to control many cell responses, and its signalling patterns are a biological conundrum [71]. Recently, NanoLuc was fused to a photoactivatable adenylate cyclase called bPAC to study how the spatiotemporal production of cAMP affects proliferation [72]. It was demonstrated that cAMP synthesis can be spatially and temporally controlled with bioluminescence in a predictable and tunable manner. This strategy allowed the systematic generation and evaluation of spatially-isolated cAMP oscillations that were decoupled from endogenous cAMP production. 

Thus, the bioluminescence-mediated regulation of proteins can simplify the design of optogenetic experiments while still maintaining a great degree of flexibility in the physiological signalling patterns that can be mimicked and studied.

But perhaps the most promising application of bioluminescence-driven optogenetics is engineering novel synthetic abilities in living organisms. Substituting external light sources with genetically encodable bioluminescence systems allows optogenetic programming of *autonomous* living organisms. A recent publication demonstrated the suitability of this approach for the development of genetic Boolean logic gates-genetic modules that endow cells with decision-making algorithms that can integrate multiple inputs and operate according to the specified logic [73,74]. An AND gate called SPARK2 was engineered based on bioluminescence resonance energy transfer between a luciferase and a light-sensor to quantitatively detect protein-protein interactions [75]. SPARK2 consists of two components: a light-switchable domain fused to a transcription factor with a peptide containing a protease-recognition site that is only exposed upon light activation and a protease fused to a luciferase. Each component is linked to a protein of interest and, if luciferin is available and the proteins of interest interact, the luciferase activates the sensing domain, exposing the cleavage site to the protease and causing the transcription factor to be released. Thus, gene expression is induced only when the two selected proteins interact “AND” the luciferase is active (Figure 2C). In the publication, this genetic logic gate was leveraged to create a high-throughput screening method for receptor agonists and to detect cell-cell interactions between two extracellular proteins.

Bioluminescence-based interactions can also be engineered to operate at a distance, without the need to physically couple proteins. Recently, an array of commonly used light sensors was systematically activated with light-emitting proteins by simply co-expressing both elements in the cell [76] (Figure 2D). It was also demonstrated that the activation pattern could be controlled by tuning the luciferin concentration.

Light-triggered events can then be further propagated to change interactions between cells or organisms. For example, a sensor for mercury was created based on bioluminescence-controlled adhesion between bacterial cells [77]. It consists of two bacterial strains expressing the *lux* operon from a mercury-sensitive promoter and photosensitive heterodimers pMagHigh or nMagHigh on the cell surface. Mercury detection induces *lux* expression, which autonomously produces bioluminescence and activates the heterodimers, leading to the formation of cell–cell adhesions. Consequently, two readouts are available for quantification of mercury: light production and cell agglutination. Moreover, cell aggregation facilitates bioluminescence detection, improving the sensitivity of the sensor, while accumulation of bacteria is less sensitive to the circuit input and allows for higher values to be detected on a different scale. Thus, cell agglutination serves as a signal amplifier for the bioluminescence readout and as a compressor for the sedimentation readout, simultaneously expanding both ends of the biosensor’s detection range.

## 5. Future Directions

As it has previously been stated, in most conditions bioluminescent proteins are “the only photonic players in an otherwise dark environment” [78]. For intracellular interactions, luciferases hold the potential to open a new synthetic dimension in the interactome decoupled from all other interactions in the cell. The proof-of-concept studies discussed in Section 4 prove the feasibility of genetically programming distinct types of molecular interactions using bioluminescence, showing that such interactions (1) can be restricted to specific subcellular locations [72,75,76]; (2) operate exclusively when protein-protein interactions occur [75]; (3) interact distantly within the cell [76]; or even (4) mediate cell-cell interactions [75,77].

Applied to cross-cell activation, bioluminescence can potentially be used as a universal life-life communication strategy where information is carried by photons, creating a species-agnostic communication interface, as envisioned in the movie Avatar [79]. For such communication, an optical synapse can be engineered, where the presynaptic cell is conditionally bioluminescent and the postsynaptic cell is capable of sensing the signal and translating it into the action potential or other physiological response (Figure 2E). Similarly, a conditionally bioluminescent cell expressing an optogenetic system may become a basis for a universal life-computer communication interface. Although light-based communication has been used to develop cell-machine interfaces to provide computer-aided regulation in real time [80,81], a truly real-time two-way optical communication between a computer and a living organism has not been achieved yet (Figure 2F). We expect that the development of bioluminescence-based optogenetics will contribute to building complexity in the way we investigate, engineer, and interface with biological systems in a predictable and modular manner.

## Figures and Tables

**Table 1:** Autonomous bioluminescence systems currently available.

Bioluminescence System	Summary
**Lux Operon from Bacteria**	The bacterial luciferase uses long-chain aldehydes and reduced flavin mononucleotide (FMNH_2_) to emit cyan light (490 nm). The bioluminescence pathway is encoded in a multicistronic lux operon that contains all the necessary genes to ensure a constant glow when transformed into other bacteria: two luciferase subunits, the three constituents of the fatty acid reductase complex, and a flavin reductase enzyme [16]. The brightest engineered version of the operon currently available is iLux [17]. While expression of the bacterial system in eukaryotic cells has been historically cumbersome [18], an improved version for mammalian cells has recently been reported [5].
**Caffeic Acid Cycle from Fungi**	The fungal bioluminescence pathway was recently elucidated, becoming the first autonomous eukaryotic system available. The fungal luciferin is 3-hydroxyhispidin, a styryl pyrone that can be produced from caffeic acid in two enzymatic steps catalysed by the hispidin synthase (HispS) and hispidin-3-hydroxylase (H3H). Fungal luciferin emits green light (520 nm) upon oxidation by the luciferase Luz, and is recycled into caffeic acid by the fourth enzyme of the pathway, caffeoyl pyruvate hydrolase (CPH) [4]. In organisms lacking caffeic acid, fungal luciferin can be produced from tyrosine, with two extra enzymatic steps [4].
